# Myopericytoma of the ureter Incidental finding of a benign slowly growing tumor

**DOI:** 10.1016/j.eucr.2023.102362

**Published:** 2023-02-21

**Authors:** Victor Sandoval, Daniel Halstuch, Melissa Huynh, Bret Wehrli, Nicholas Power

**Affiliations:** a Western University, London Health Science Center (LHSC), 151 Richmond St, London, ON, N6A 3K7, Canada; b Western University, University Hospital (UH), 151 Richmond St, London, ON, N6A 3K7, Canada

**Keywords:** Tumor, Myopericytoma, Benign, Ureter

## Abstract

We present a unique case of a 6 cm, incidental, ureteral myopericytoma which was initially believed to be an ovarian tumor with mass effect, causing hydroureteronephrosis. A 75-year-old woman presented with a three-month history of postprandial cramps and heartburn. A right distal ureterectomy with en-bloc resection of the mass was performed.

Histologically, a well-circumscribed, cellular proliferation of uniform, cytologically bland, spindle cells was identified that had a multilayered, concentric growth pattern around numerous blood vessels. Immunohistochemically, the spindle lesional cells stained strongly and diffusely with antibodies against smooth muscle actin and failed to stain for pancytokeratin and S100 protein.

## 1 Introduction

Myopericytoma is a rare, benign, mesenchymal neoplasm thought to be derived from myopericytes which are transitional cells with overlapping features of pericytes and vascular smooth muscle cells.^1^ Although myopericytomas most commonly arise in the skin and subcutis of the distal and proximal extremities, followed by the trunk, and head and neck regions,^2^ rarely, these tumors can occur in deep soft tissue and visceral sites. Several case reports and small series of myopericytomas involving the urinary tract, including the kidney, bladder and renal pelvis, have been published.^3^^,^^4^ The World Health Organization recognized myopericytoma as a distinct entity in its 2013 classification of soft tissue tumors. They are classified as part of the pericytic (perivascular) tumor family which also includes myofibroma, angioleiomyoma and glomus tumor.^5^ Histologically, these tumors are characterized by proliferations of perivascular round to spindle-shaped, cytologically bland tumor cells with amphophilic to lightly eosinophilic cytoplasm. We describe here a case of a benign myopericytoma involving the right ureter.

## 2 Case presentation

**Fig. 1:**
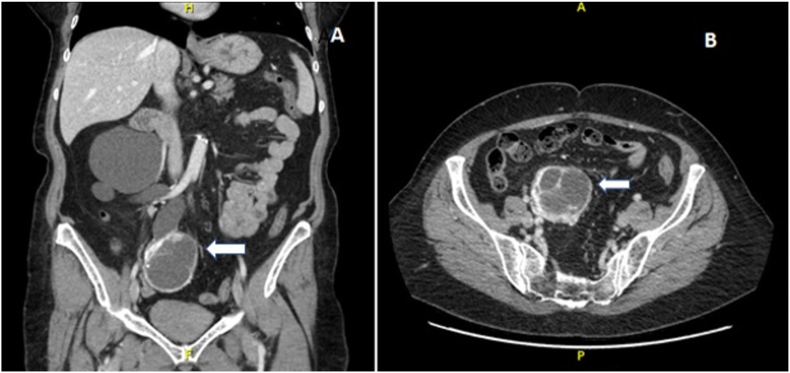
A. CT abdomen with contrast, coronal view. **B.** CT of the abdomen with contrast, axial view. Redemonstration of severe right hydronephrosis and hydroureter down to the level of the mid ureter, secondary to obstruction from what appeared to be a right ovarian mass.

**Fig. 2:**
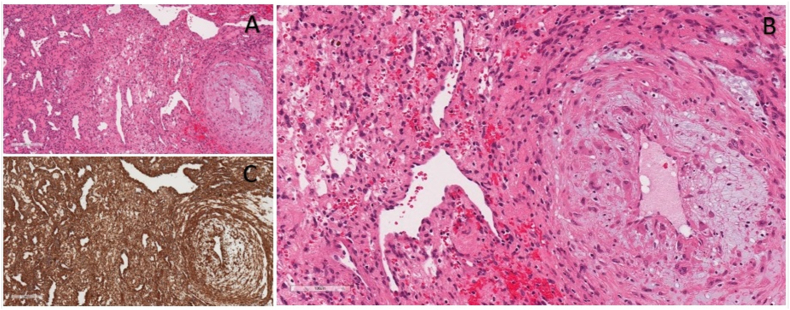
A. H&E 5× magnification moderately cellular, oval to spindle cell proliferation with a prominent perivascular, concentric growth pattern and background hemangiopericytomatous vasculature is evident at low magnification **B.** H&E 20× high magnification image, the lesional cells are uniform, lack significant cytological atypia and mitotic activity **C.** Smooth Muscle Actin immunohistochemical staining 10× Diffuse, strong, cytoplasmic immunostaining of the lesional cells for smooth actin is present.

A 75-year-old woman presented with a three-month history of postprandial cramps and heartburn. She underwent a computed tomography scan of her abdomen and pelvis, which revealed an incidental, 6.7 × 5.8 × 5.3 cm, enhancing pelvic mass thought to be originating from the right ovary, with associated right-sided severe hydronephrosis and a dilated tortuous right ureter (Fig. 1). There was no evidence of local lymphadenopathy or metastasis. The patient had no dysuria or gross hematuria. Her relevant past surgical history included a transabdominal hysterectomy with left salpingo-oophorectomy. She was referred to gynecologic oncology for further assessment and subsequently underwent an exploratory laparotomy for a possible right salpingo-oophorectomy. However, intraoperative exploration revealed that the mass was actually arising from the right ureter. At this point, the Urology team joined the procedure, and a right distal ureterectomy with en-bloc resection of the mass was performed. Reconstruction included a ureteroneocystostomy with a psoas hitch. The surgery was uneventful, and the patient was discharged on post-operative day 5. On the pathological report macroscopically, the ureter measured 15.7 cm in length, had a luminal circumference of up to 6.0 cm, and periureteral tissue of up to 3.3 cm depth. The right ovary and fallopian tube were unremarkable. Histologically, a well-circumscribed, cellular proliferation of uniform, cytologically bland, spindle cells was identified that had a multilayered, concentric growth pattern around numerous blood vessels. A prominent background hemangiopericytomatous vascular pattern was present in areas (Fig. 2). Mitotic activity was inconspicuous. Urothelium and adjacent smooth muscle were identified in keeping with entrapped ureter. Epithelial dysplasia and malignancy were not identified. Immunohistochemically, the spindle lesional cells stained strongly and diffusely with antibodies against smooth muscle actin and failed to stain for pancytokeratin, S100 protein, desmin, CD34, Stat6, ERG and CD31. Antibodies against Melan-A and HMB45 were both negative. The absence of staining for these two antibodies argues against a diagnosis of Pecoma. Morphological and immunohistochemical features were those of a myopericytoma arising from the ureteric tissues.

## 3 Discussion

We present a unique case of a 6 cm, incidental, ureteral myopericytoma which was initially believed to be an ovarian tumor. To our knowledge this is the first case report of a primary myopericytoma originating from the ureter.

Myopericytomas have a well-defined immunohistochemical staining pattern in that they are positive for vimentin, smooth muscle actin, and caldesmon, but negative or only focally positive for desmin and CD34. Our case stained identically to the 6 renal myopericytomas described by Li et al.^2^

Most patients with myopericytomas involving the urinary tract are asymptomatic. The reported cases involving the kidney usually presented as an asymptomatic solitary renal mass. The patients had a mean age of 46 (range 17–76 years). The urinary tract myopericytomas ranged in size from 1.7 cm to 11.0cm.^4^ The main diagnostic consideration in most cases was a renal cell carcinoma. Gross hematuria was the initial presentation in one patient with a myopericytoma arising from the renal cortex extending into the ureter^3^ and in another patient with a myopericytoma arising from the trigone of the bladder, which prevented spontaneous passage of a ureteral calculus.^4^ Hematuria in the latter case was attributed to the impacted stone. In our case, the mass was diagnosed incidentally after abdominal cramps, possibly secondary to mass effects. Hematuria and hypertension were not reported. On initial imaging, a CT scan of the kidneys demonstrated right hydronephrosis with right hydroureter down to the level of the mid-ureter. The images suggested ureteric obstruction from a complex, solid and cystic, right ovarian mass. An MRI of the pelvis showed a multilocular, cystic, right-sided, pelvic mass that was concerning for a right ovarian malignancy.

## 4 Conclusion

Myopericytomas are benign, slow-growing, perivascular tumors that are mostly found in the skin and subcutaneous tissues. There are limited case reports of this tumor in the urinary tract, primarily in the kidneys. Because this tumor presents with very few symptoms, a high level of suspicion is needed to consider this entity in the differential diagnosis for vascular lesions found on imaging of the genitourinary tract. To the best of our knowledge, no recurrences or metastases of myopericytomas originating from the urinary tract have been reported.

## References

[1] Lau S.K., Klein R., Jiang Z., Weiss L.M., Chu P.G. (2010 Oct). Myopericytoma of the kidney. Hum Pathol.

[2] Li J., Zhao M., Chen Z., Zou L., Teng X. (2015). Renal myopericytoma: a clinicopathologic study of six cases and review of the literature [Internet]. Int J Clin Exp Pathol.

[3] Budding L., Rothman S.F., Goedhals J. (2020 Nov). Myopericytoma involving the right renal pelvis and ureter: a case study. Hum Pathol: Case Rep.

[4] Sirohi D., Smith S.C., Epstein J.I. (2017 Jun). Pericytic tumors of the kidney—a clinicopathologic analysis of 17 cases. Hum Pathol.

[5] Mangham D.C. (2004 Apr). World Health Organisation classification of tumours: pathology and genetics of tumours of soft tissue and bone. J Bone Joint Surg British.

